# *B. infantis* EVC001 Is Well-Tolerated and Improves Human Milk Oligosaccharide Utilization in Preterm Infants in the Neonatal Intensive Care Unit

**DOI:** 10.3389/fped.2021.795970

**Published:** 2022-01-05

**Authors:** Sarah Bajorek, Rebbeca M. Duar, Maxwell Corrigan, Christa Matrone, Kathryn A. Winn, Susan Norman, Ryan D. Mitchell, Orla Cagney, Alexander A. Aksenov, Alexey V. Melnik, Evguenia Kopylova, Jose Perez

**Affiliations:** ^1^St. Mary's Hospital, Grand Junction, CO, United States; ^2^Orlando Health Winnie Palmer Hospital for Women and Babies, Orlando, FL, United States; ^3^Evolve BioSystems Inc., Davis, CA, United States; ^4^Department of Chemistry, University of Connecticut, Storrs, CT, United States; ^5^Arome Science Inc., Farmington, CT, United States; ^6^Clarity Genomics Inc., San Diego, CA, United States; ^7^Seattle Children's Hospital, University of Washington, Seattle, WA, United States

**Keywords:** preterm, *B. infantis*, microbiome, probiotics, human milk

## Abstract

Not all infants carry specialized gut microbes, meaning they cannot digest human milk oligosaccharides and therefore do not receive complete benefits from human milk. *B. infantis* EVC001 is equipped to convert the full array of complex oligosaccharides into compounds usable by the infant, making it an ideal candidate to stabilize gut function and improve nutrition in preterm infants. A prospective, open-label study design was used to evaluate the tolerability of *B. infantis* EVC001 and its effects on the fecal microbiota in preterm infants in a Neonatal Intensive Care Unit. Thirty preterm infants <1,500 g and/or <33 weeks gestation at birth were divided into two matched groups, and control infants were enrolled and discharged prior to enrolling EVC001 infants to prevent cross-colonization of *B. infantis*: (1) fifteen control infants received no EVC001, and (2) fifteen infants received once-daily feedings of *B. infantis* EVC001 (8.0 x 10^9^ CFU) in MCT oil. Clinical information regarding medications, growth, nutrition, gastrointestinal events, diagnoses, and procedures was collected throughout admission. Infant stool samples were collected at baseline, Study Days 14 and 28, and 34-, 36-, and 38-weeks of gestation. Taxonomic composition of the fecal microbiota, functional microbiota analysis, *B. infantis*, and human milk oligosaccharides (HMOs) in the stool were determined or quantified using 16S rRNA gene sequencing, metagenomic sequencing, qPCR, and mass spectrometry, respectively. No adverse events or tolerability issues related to EVC001 were reported. Control infants had no detectable levels of *B. infantis*. EVC001 infants achieved high levels of *B. infantis* (mean = 9.7 Log10 CFU/μg fecal DNA) by Study Day 14, correlating with less fecal HMOs (ρ = −0.83, *P* < 0.0001), indicating better HMO utilization in the gut. In this study, *B. infantis* EVC001 was shown to be safe, well-tolerated, and efficient in colonizing the preterm infant gut and able to increase the abundance of bifidobacteria capable of metabolizing HMOs, resulting in significantly improved utilization of human milk.

**Clinical Trial Registration:** https://clinicaltrials.gov/ct2/show/NCT03939546, identifier: NCT03939546.

## Introduction

The initial colonization of the neonatal gut plays a critical role in overall gut microbiota assembly, which can affect gut and systemic health across the lifespan (1). Increasing evidence strongly suggests the presence of specific members of the genus *Bifidobacterium* play an important role during early human development (2). Products of bifidobacterial metabolism in the infant gut have been discovered to be essential mediators in the development of the immune system (3) and create biochemical changes in the gut lumen, deterrent to pathogen invasion and expansion (4, 5).

Gestational age is a main factor that influences the assembly of the neonatal gut microbiota (6). Preterm infants born <32 weeks gestational age are anatomically and immunologically underdeveloped, and more likely to be born by caesarean section, disrupting the mother-to-infant transmission of gut microorganisms and delaying colonization with beneficial bifidobacteria (7–9). In addition, hospitalized preterm infants in the neonatal intensive care unit (NICU) are colonized by potential pathogens found on hospital surfaces (6, 10) and are regularly exposed to medications such as antimicrobials (11). These circumstances have a profound and detrimental impact on the developing preterm gut microbiome, including a propensity for high-mortality conditions such as necrotizing enterocolitis (NEC) and late onset sepsis (12). Thus, strategies to stabilize and support overall gut health by increasing populations of beneficial *Bifidobacterium* in the preterm gut warrant attention.

The formation of a *Bifidobacterium*-dominant gut microbiome in neonates is attributed to the presence of human milk oligosaccharides (HMOs), which are the third most abundant solid component in human milk after lactose and lipids (13–15). *Bifidobacterium longum* subspecies *infantis* (*B. infantis*) is an infant gut symbiont equipped to utilize the full array of HMOs in breast milk (16). Colonization of *B. infantis* EVC001 in term infants fed an exclusive human milk diet has been shown to increase the abundance of bifidobacteria and reduce taxa capable of eliciting pathogenicity (17). Although *B. infantis* EVC001 has been previously fed to preterm infants with no adverse effects (18), this open-label study aimed specifically to prospectively evaluate the safety and tolerability of *B. infantis* EVC001 and its effects on the fecal microbiota in at-risk, hospitalized preterm infants (<33 weeks gestational age at birth or <1,500 g birth weight) in the NICU at Orlando Health Winnie Palmer Hospital. We hypothesized that *B. infantis* EVC001 would be tolerated by preterm infants in the NICU, based on this bacterium being an infant-adapted microbe. Additionally, we hypothesized that infants in the EVC001 group would have significantly more *B. infantis* and less HMOs in their stool than their control counterparts, suggesting more complete nutrient utilization in the gut by the probiotic bacteria.

## Materials and Methods

### Study Design

This was a single-center, open-label, prospective study of an infant probiotic (activated *B. infantis* EVC001, a Food for Special Dietary Use) in preterm infants, conducted in the NICU at Orlando Health Winnie Palmer Hospital for Women & Babies. The study protocol was reviewed and approved by the Orlando Health Institutional Review Board. The rationale for not conducting the study as a double-blind, randomized, placebo-controlled trial (RCT) is based on literature from previous probiotic RCTs in the NICU environment where infants in the placebo group incidentally acquired the probiotic bacteria (19, 20). Additionally, since the study was not blinded, medium chain triglyceride (MCT) oil, the carrier in the *B. infantis* product, was not fed to infants in the control group in order to minimize unnecessary intervention.

**Figure 1:**
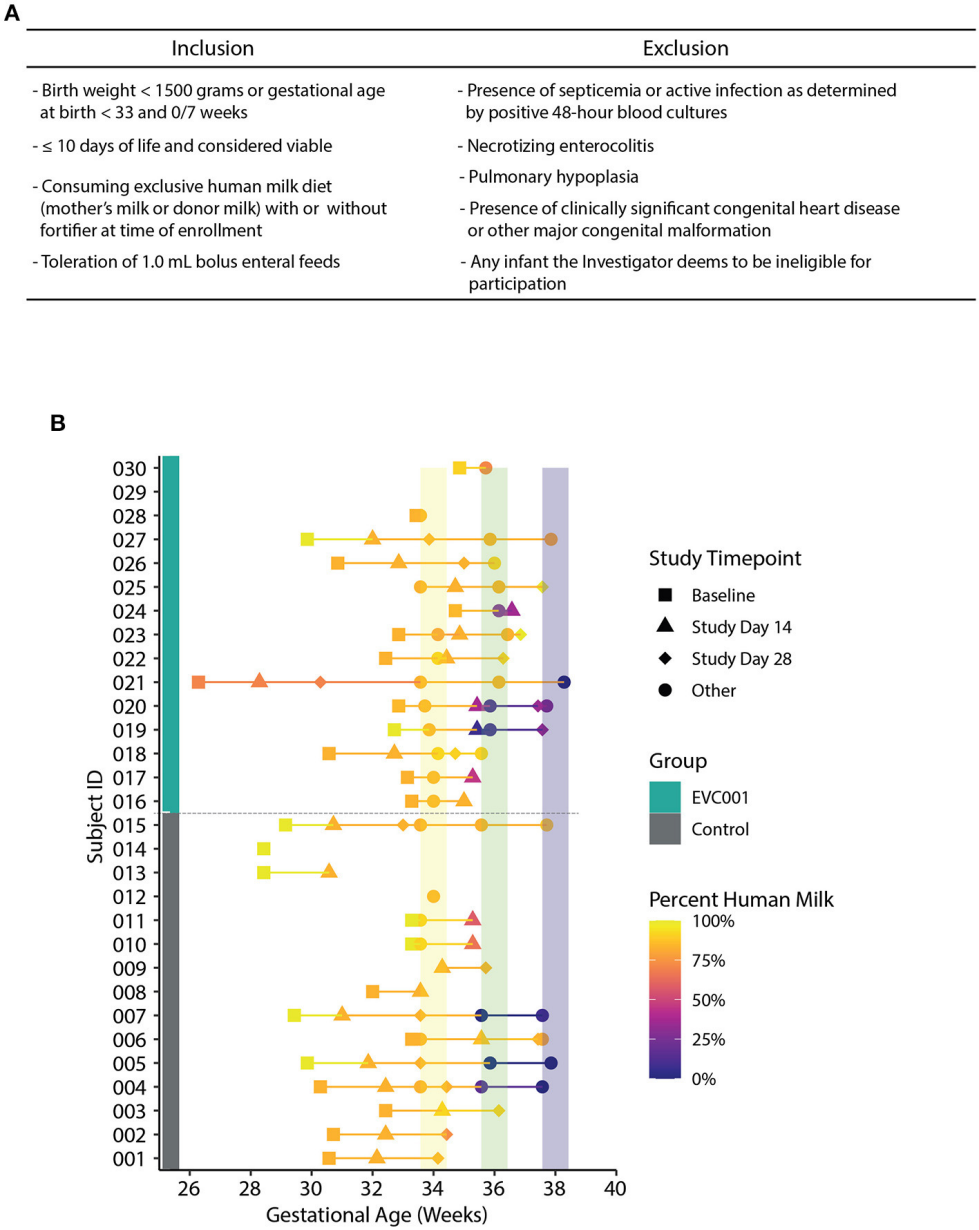
**(A)** Inclusion and inclusion criteria for participation in the study. **(B)** Fecal samples collected over time, arranged by Study ID (y-axis) and corrected gestational age (x-axis). Group assignment is denoted by a teal (EVC001) or gray box (control). The data points are shaded by the abundance of human milk an infant was receiving at sample collection and shapes represent study day for sample collection (see legend). Colored vertical lines represent interval periods for sample collection at targeted gestational age (+/- 3 days).

The target participants were male and female premature NICU inpatients, weighing < 1,500 grams at birth or < 33 weeks gestational age at birth, delivered vaginally or by cesarean section. Full inclusion and exclusion criteria can be found in Figure 1A. Study investigators met with the parent(s) of eligible participants to facilitate informed consent. No study-related activities were performed on any participant prior to parental consent.

All baseline activities (data and stool collection) occurred prior to Day 10 of life in order for infants to be eligible for participation. Study Day 0 was designated as the day that all baseline activities were complete (for both groups) and the day of the first *B. infantis* EVC001 feeding (for the EVC001 group). Fifteen infants were initially enrolled into the control group and completed their hospital stay, after which 15 infants were enrolled into the EVC001 group. Infants enrolled into the control group received no supplemental study product or placebo. Infants enrolled in the EVC001 group received a daily feeding of 8.0 x 10^9^ CFU of activated *B. infantis* EVC001 (commercially available as Evivo® in MCT oil, Evolve BioSystems, Inc.) initiated by day 10 of life and continued until hospital discharge. The form of *B. infantis* was selected because it is a small volume of liquid in a single-serve vial delivering 8.0 x 10^9^ CFU in 0.5 mL of MCT oil, a carrier with a long history of use in preterm infants. *B. longum* subsp. *infantis* EVC001 was selected for this study because of its demonstrated ability to consume HMO from human milk (17, 21). EVC001 was first isolated from a healthy term breastfed infant and has been well-characterized (21). The product vials were stored frozen in the pharmacy and dispensed weekly to the NICU in designated study refrigerators where nurses could access each infant's daily vial, sufficiently warm the vial, withdraw the contents (0.5 mL of MCT oil and *B. infantis* EVC001), and deliver the product as trained. The study product was provided via nasogastric, orogastric, or gastrostomy tube or fed orally for infants who were no longer tube-fed. EVC001 feedings were held if infants were NPO (nothing by mouth), and feeds resumed in tandem with reintroduction of PO (by mouth) feeding. Feeding of EVC001 continued during oral and IV antibiotic courses.

### Data Collection

Any medical event or diagnosis occurring prior to Study Day 0 was collected as Baseline Clinical Data and not as an adverse event (AE). Anthropometric data was collected weekly from the medical records as measured for standard of care. Only specific categories of concomitant medications were collected for the study: caffeine, systemic antibiotics, antifungals, H2 blockers/proton pump inhibitors (PPI), enemas/suppositories, and motility medications. Number of stools was documented from the medical record daily. The following gastrointestinal (GI)-related events were included as data points when they required treatment, diagnostics, or a change in standard of care: blood in stool, abdominal distension, emesis, abnormal gastric residuals, and diaper rash. Information about the infant's nutritional intake (maternal breast milk, donor breast milk, human-derived human milk fortifier, bovine human milk fortifier, infant formula) and feeding mode [intravenous/total parenteral nutrition (IV/TPN), nasogastric/orogastric (NG/OG), by mouth (PO)] were captured weekly, as well as at each stool collection timepoint. Prior to the start of the study, an Adverse Event Reporting Plan was developed regarding the documentation of adverse events and specified that select medical events that are typically expected in this patient population would not be reported as AEs. The list included: anemia, apnea, desaturation, bradycardia spells, bronchopulmonary dysplasia, chronic lung disease, intraventricular hemorrhage, hypoglycemia, hyponatremia, hypothermia, impaired tissue integrity that does not require treatment, irregular blood pressures or heart rates that do not result in treatment or change in health status, jaundice, neutropenia, patent ductus arteriosus, retinopathy of prematurity, suspected sepsis or infectious screen that does not require antibiotic treatment, and thrombocytopenia. These events were included as data points specific to hospital course along with total days of TPN, time to full feeds (150 mL/kg/day), blood product transfusions, and respiratory support.

### Stool Sample Collection

Infant stool specimens were collected during six timepoints throughout the course of the study; baseline, Study Days 14 and 28, and 34-, 36-, and 38-weeks gestational age (Figure 1B). A single swab was collected and placed into lysis/stabilization buffer at all timepoints (described below). A total of 14 baseline stool swabs were collected from control infants (5 with meconium present) and 13 from EVC001 infants (2 with meconium present). At Study Day 14 (control *n* = 13 and EVC001 *n* = 12) and 34 weeks gestational age (control *n* = 13 and EVC001 *n* = 11), an additional undiluted stool sample was collected into a 5 mL snap-cap tube. A 7-day collection window was permitted for each collection timepoint. Stool specimens were not collected after hospital discharge. If a stool timepoint fell during a time when the infant was receiving oral or IV antibiotics, an additional sample was collected 1 week after the last day of that course of antibiotics. Fecal samples were collected from the diaper by the study personnel and immediately transported on ice to the hospital's biorepository and stored at −80°C. Samples were shipped upon completion of each of the study groups on dry ice to the laboratory where they were stored at −80°C for subsequent analysis.

### Fecal DNA Extraction and Microbiota Studies

DNA was extracted from stool swab samples stored in DNA/RNA shield lysis tubes (Zymo Research, Irvine, CA) using the ZymoBIOMICS 96 MagBead DNA kit (Zymo Research). Extracted DNA was quantified using QuantIT dsDNA Assay kit, high sensitivity (Thermo Fisher Scientific, Waltham, MA) according to the manufacturer's protocol.

Libraries were prepared from Study Day 14 samples for shotgun metagenomic sequencing using the Illumina DNA Prep Kit with unique dual indexes (Illumina, San Diego, CA, USA). Libraries were pooled to equimolar ratios and submitted to UC Davis DNA Technologies Core for sequencing on an Illumina NovaSeq S4 flow cell (Illumina, San Diego, CA). Additionally, all samples were subjected to 16S ribosomal RNA (rRNA) gene sequencing as previously described (17). Demultiplexed shotgun metagenomic reads were trimmed and human read filtered using trimmomatic v.33 (22) and bowtie2 v2.4.2 (23) as part of the Kneaddata pipeline v0.10.0 (24). Taxonomic and strain profiling was performed as previously described (25). Taxonomic profiling of the metagenomic samples was performed using MetaPhlAn2 (26), which uses a library of clade-specific markers to provide pan microbial (bacterial, archaeal, viral, and eukaryotic) profiling (http://hutten-hower.sph.harvard.edu/metaphlan2), in combination with HUMAnN2 (27) to profile functional metagenomics against Uniref90 following the updated global profiling of the Human Microbiome Project (2017). Cross database annotations (e.g., UniProt to KEGG) were performed within HUMAnN2 using the “utility_mapping” conversion tool package. Functional HMO utilization gene profiles were generated with HUMAnN2 bypassing all steps except “nucleotide-search” using a custom nucleotide database of HMO genes. Gene-level reads per kilobase (RPK) values were then renormalized to copies per million (CPM) units to control for gene length distribution across samples using “humann2_renorm_table.” A heatmap of HMO utilization genes using CPM values was generated in R (v 4.0.4) using tidyheatmap package (v0.0.0.9).

16S gene rRNA sequences were analyzed within the QIIME2 (release 2020.2) (28) and R (version 4.0.4). Paired end sequences were demultiplexed. Low-quality ends of the reads were trimmed, and the reads were joined and denoised with DADA2 (29) implemented in the q2-dada2 plugin. Low-quality ends of the reads were trimmed, and the reads were joined and denoised with DADA2 (29) implemented in the q2-dada2 plugin. Taxonomy was assigned to each feature using a pretrained Naive Bayes classifier against the Greengenes 3_8 99% OTUs databases using via the q2-feature-classifier tool. The feature table was collapsed to the family level. Inter and intra treatment group differential abundance analysis was performed using q2-Aldex2 (30–32) plugin on the CLR-transformed family level feature table. Phylogenic alignments were conducted with mafft (33) and fasttree (34) and Shannon Diversity and Weighted Unifrac distance matrix (35) were created using the q2-diversity plugin in qiime2, rarified to the minimum number of dereplicated sequences passing quality filter for the samples (15,000 reads per sample). Shannon diversity values were compared at specific study days using a Wilcoxon Rank Sum test and graphed using ggpubr (v0.4.0) and ggplot2 (v3.3.5) in R. Analysis of variance using distance matrices (adonis) was used to compare beta diversity between groups. Additionally, binary Jaccard distances were used to assess compositional changes between the groups over time in a paired sample approach using the q2-longitudinal package within QIIME2 (36).

### Absolute Quantification of Fecal *B. infantis* by Real-Time PCR

Total *B. infantis* was quantified in fecal samples from infants at Study Day 14 and Study Day 28. Quantification was performed by real-time PCR using the Blon_2348 sialidase gene primers (37) and conducted as previously described (8). Absolute quantification was determined using a standard curve prepared using 10-fold dilution series of genomic DNA derived from a pure liquid culture of *B. infantis* EVC001 for which the CFU counts had been determined by quantitative plating. Quantities obtained were Log 10 converted and normalized to total stool DNA to yield Log10 CFU of *B. infantis* /μg of fecal DNA.

### Absolute Quantification of HMOs in Fecal Samples

Absolute quantification of HMOs was performed in fecal samples obtained from infants at Study Day 14 using high performance liquid chromatography mass spectrometry (HPLC-MS). Fecal samples were kept on ice for processing and diluted to 1 mg/10 μl with the Extraction Solvent (LC-MS grade water + mix of internal standards 5C13-Glu and internal standard, Sulfachloropyridazine at 50uM) in a clean microcentrifuge tube. Tubes were sealed with parafilm, and samples were sonicated for 5 min in ice water, followed by homogenization on a shaker for 2 h at 4°C, then centrifuged at ~17,530 x g for 10 min to obtain the “Original Supernatants.” These samples were further processed with solid phase extraction (SPE) for LC-MS analysis using Oasis HLB SPE cartridges (1cc Vac RC Cartridge 30 mg Sorbent 30um, Waters, Milford, MA, USA). Standards of 2′-fucosyllactose (2′-FL), 3-fucosyllactose (3′-FL), lacto-*N*-tetraose (LNT), and 6′-Sialyllactose (6′-SL) were purchased from Carbosynth Ltd Kingsclere, Newbury, England. Lacto-*N*-fucopentaose I (LNFP I) was obtained from Boca Scientific, Dedham, MA, USA. These 5 standards have previously been used to determine a universal response factor to estimate the total oligosaccharide content in milk due to their consistent presence across samples and correlation to the full glycan load (38–40). Calibration curves were prepared as previously described (40). The HILIC data acquisition was conducted on a Thermo Vanquish Horizon uHPLC with Thermo Orbitrap Elite Mass Spectrometer with ESI source (Thermo Scientific, Waltham, MA, USA) using a Waters BEH amide HILIC UHPLC column. Column compartment was maintained at 50°C using forced air. Solvent A: Water + 0.1 % FA; solvent B: ACN + 0.1 % FA. MS spectra were acquired in negative ion mode in the range of 50-2000 m/z. The instrument settings were as follows: Spray voltage: - 2.5kV; Cap temp: 300°C; Sheath gas: 47.5 L/min; Aux gas: 11.25 L/min; Spare Gas: 2.25 L/min; Max spray current: 100; Probe heater 412.5; S-lens: 50 V. The chilled compartment temperature was maintained throughout the analysis at 10°C (38, 39).

### Sample Size

The study sample size was calculated based on historical sample sizes of similar and comparable clinical tolerability trials investigating the use of probiotics and food products (41–44). Power calculations were performed for the serving size required to increase fecal *B. infantis* levels compared to a control group in exclusively breastfed infants. Based on infant fecal *B. infantis* levels from a previous study of *B. infantis* EVC001 (17), 4 infants would be required in each group to identify a 9.7-log CFU difference, with an α = 0.01 (to account for multiple testing within each family of hypotheses) and power = 90%. Given that the observed variability in a study with only 4 infants per group could be inflated due to a single outlier, a minimum of 10 infants in each group was selected to reduce the impact of a potential outlier. Furthermore, to account for a 30% attrition rate, 15 infants were selected for enrollment in each group.

### Statistical Analyses

Statistical analysis was performed using R version 4.0.4. Means and standard errors (SE) were calculated for bar charts of continuous data. Two-sided Wilcoxon Rank Sum test was used for statistical comparisons of continuous variables. A chi-square test, or Fisher's exact test for small counts, was used for comparison of categorical variables. Spearman's correlations with Benjamini-Hochberg false discovery rate (FDR) correction were run for relationships between continuous variables. Beta diversity between the treatment groups and the effect size was determined using permutational multivariate analysis of variance using distance matrices (adonis) on the weighted unifrac distance matrix between groups. Additional beta-diversity differences between groups using a linear mixed model was used to assess changes in binary Jaccard distance over time. *P*-values < 0.05 were considered significant.

## Results

### Recruitment, Demographics, and Baseline Clinical Data

Thirty preterm infants < 1,500 grams at birth or < 33 weeks gestational age at birth from Orlando Health Winnie Palmer Hospital for Women & Babies were enrolled in this study from July 2019 to April 2021 (Supplemental Figure 1). At enrollment, the average gestational age was 30.3 and 31.3 weeks for infants in the control and EVC001 groups, respectively (*P* = 0.15). The average birth weight was lower in the control group (1,372 grams) but not statistically different (*P* = 0.09) compared to the EVC001 group (1,564 grams). Race and ethnicity were statistically significant between groups (*P* = 0.01) with a higher percent of white infants in the EVC001 group and a higher percent of Hispanic or Latino infants in the control group (*P* = 0.01). Additionally, on average, infants in the EVC001 group had significantly fewer (*P* = 0.04) days on TPN (2.7 days) compared to control infants (4.6 days). All other characteristics (birth mode, multiple birth, maternal antibiotics at delivery, and infant sex) were not significantly different between groups (Table 1). Baseline clinical data was collected prior to Study Day 0 for both groups and is reported in Supplemental Table 1. Nutritional intake (percent of diet that was human milk) at the time of each stool sample collection was similar between groups (*P* = 0.605) and is presented in Figure 1B.

**Table 1:** Subject Characteristics.

		**Control group (*****n*** **= 15)**		**EVC001 group (*****n*** **= 15)**			
		**Mean (min, max)**		**Mean (min, max)**		***P*** **-value**	**Statistical test**
Birth weight (grams)		1,372 (920, 2080)		1,564 (720, 2,340)		0.09	Wilcoxon
Gestational age at birth (weeks)		30.3 (28.0, 32.7)		31.3 (25.1, 34.0)		0.15	Wilcoxon
TPN requirement (days)		4.6		2.7		0.04	Wilcoxon
		**Count**	**Percentage**	**Count**	**Percentage**	***P*** **-value**	**Statistical test**
**Race of baby**							
	Black or African American	4	27%	3	20%	0.01	Fisher's exact
	Other	10	67%	2	13%		
	White	1	7%	9	60%		
	Asian	0	0%	1	7%		
**Ethnicity of baby**							
	Hispanic or Latino	10	67%	3	20%	0.01	Chi-squared
	Not Hispanic or Latino	5	33%	12	80%		
**Sex of baby**							
	Female	7	47%	11	73%	0.14	Chi-squared
	Male	8	53%	4	27%		
**Birth mode**							
	Cesarean section	10	67%	13	87%	0.20	Fisher's exact
	Vaginal	5	33%	2	13%		
**Delivery**							
	Singleton	8	53%	10	67%	0.46	Chi-squared
	Multiple	7	47%	5	34%		
**Maternal antibiotics at delivery**							
	Yes	10	67%	10	67%	1.00	Chi-squared
	No	5	33%	5	34%		

### Safety and Tolerability

Infants in the EVC001 group were fed the study product daily, starting on average by day of life 9, receiving between 9 and 100 study product feeds. The average number of study product feeds per infant in the EVC001 group was 36, with 11 of the 15 infants having no missed study product feedings. There were 2 infants who missed 1 day of the study product due to NPO and there were 2 infants who missed 1 day of the study product due to a protocol deviation (missed feeds by nursing staff).

There were no significant differences between the EVC001 and control groups in the number of infants with respect to any individual adverse events or serious adverse events. Two deaths occurred in subjects in the control group and zero in the EVC001 group, and infectious adverse events were higher in the control group than the EVC001 group (33% vs. 7%) but these did not reach statistical significance (*P* = 0.17). Zero cases of probiotic sepsis were reported in the EVC001 group (Table 2). Other data collected from the discharge summary of inpatient stay is summarized in Table 3. There were 6 infants in the control group who had anemia in the post-baseline period reported in the discharge summary, a statistically significant difference from the EVC001 group with zero anemia diagnoses post-baseline (*P* = 0.02).

**Table 2:** Safety and Tolerability.

		**Control group (*****n*** **= 15)**		**EVC001 group (*****n*** **= 15)**		
		**Count**	**Percentage**	**Count**	**Percentage**	***P*-value^†^**
Adverse event analysis^§^	**Any adverse event**	6	40%	6	40%	1.00^⁑^
	Any Serious Adverse Event	2	13%	2	13%	1.00
	Serious Adverse Event resulting in death	2	13%	0	0%	0.48
	Probiotic sepsis	0	0%	0	0%	1.00
	Infectious Adverse Event	5	33%	1	7%	0.17
	IV antibiotics for infection/suspected infection	3	20%	0	0%	0.22
	Prophylactic IV antibiotics for surgery	0	0%	1	7%	1.00
Adverse event counts and severity (number of events)	**Number of adverse events**	15		8		
	Number of Serious Adverse Events	4		2		0.65
	Mild	7		3		0.25
	Moderate	4		3		1.00
	Severe	1		2		1.00
	Life-threatening	1		0		1.00
	Death	2		0		0.48
GI-related surgeries or procedures^§^	**Any GI-related surgery or procedure**	0	0%	1	7%	1.00
	Endoscopy	0	0%	0	0%	1.00
	Fluoroscopy	0	0%	0	0%	1.00
	Laparoscopy	0	0%	0	0%	1.00
	Bowel resection	0	0%	0	0%	1.00
	Colostomy	0	0%	0	0%	1.00
	G-tube placement	0	0%	1	7%	1.00
	Swallow study under fluoroscopy	0	0%	1	7%	1.00
GI-related events^§^	**Any GI-related event**	3	20%	2	13%	1.00
	Visualized frank blood in stool	1	7%	0	0%	1.00
	Abdominal distention	1	7%	1	7%	1.00
	Significant or bilious emesis	0	0%	1	7%	1.00
	Disruption in skin integrity of diaper area	2	13%	0	0%	0.48
	Gastroesophageal reflux	0	0%	2	13%	0.48

§ *Counts represent the number of infants with the indicated event or procedure*.

† *Fisher's exact test*.

⁑ *Chi-squared test*.

**Table 3:** Discharge Summary.

	**Control group (*n* = 15)**	**EVC001 group (*n* = 15)**	
**Post-baseline diagnoses**	***n***	***n***	***P*-value^†^**
Anemia	6	0	0.02
Apnea, desaturation, bradycardia spells	12	11	1.00
Bronchopulmonary dysplasia / chronic lung disease	0	1	1.00
Hydrocele, non-congenital	0	1	1.00
Hyperbilirubinemia	2	0	0.48
Hypernatremia	0	1	1.00
Hypocalcemia	0	1	1.00
Hypophosphatemia	0	1	1.00
Intraventricular hemorrhage	1	0	1.00
Neutropenia	1	0	1.00
Retinopathy of prematurity	0	1	1.00
**Nutrition / Respiratory / Blood products**			
Any maternal milk	15	15	1.00
Any donor milk	15	15	1.00
Any infant formula	7	7	1.00^⁑^
Any TPN	15	14	1.00
Any blood product transfusions	1	1	1.00
Any days on ventilator	4	2	0.65

† *Fisher's exact test*.

⁑ *Chi-squared test*.

The rate of weight gain and achieved weight, length, and head circumference were not significantly different between the EVC001 and control groups (Supplemental Table 2). Stooling patterns differed significantly between control and EVC001 groups. The average number of stools per day during the baseline period was 2.46 and 3.64, respectively (*P* = 0.0003) and post-baseline was 4.48 and 2.26, respectively (*P* = 0.002) (Supplemental Table 2), indicating a significant increase in the number of stools per day in the control group compared to a significant decrease in the EVC001 group.

Use of concomitant medications collected for the study was not significantly different between groups (Supplemental Table 3). Importantly, as it relates to observed differences in stool numbers, infants in the EVC001 group did not require more enemas, suppositories, or other motility drugs than the control group.

### Microbiota Analysis

**Figure 2:**
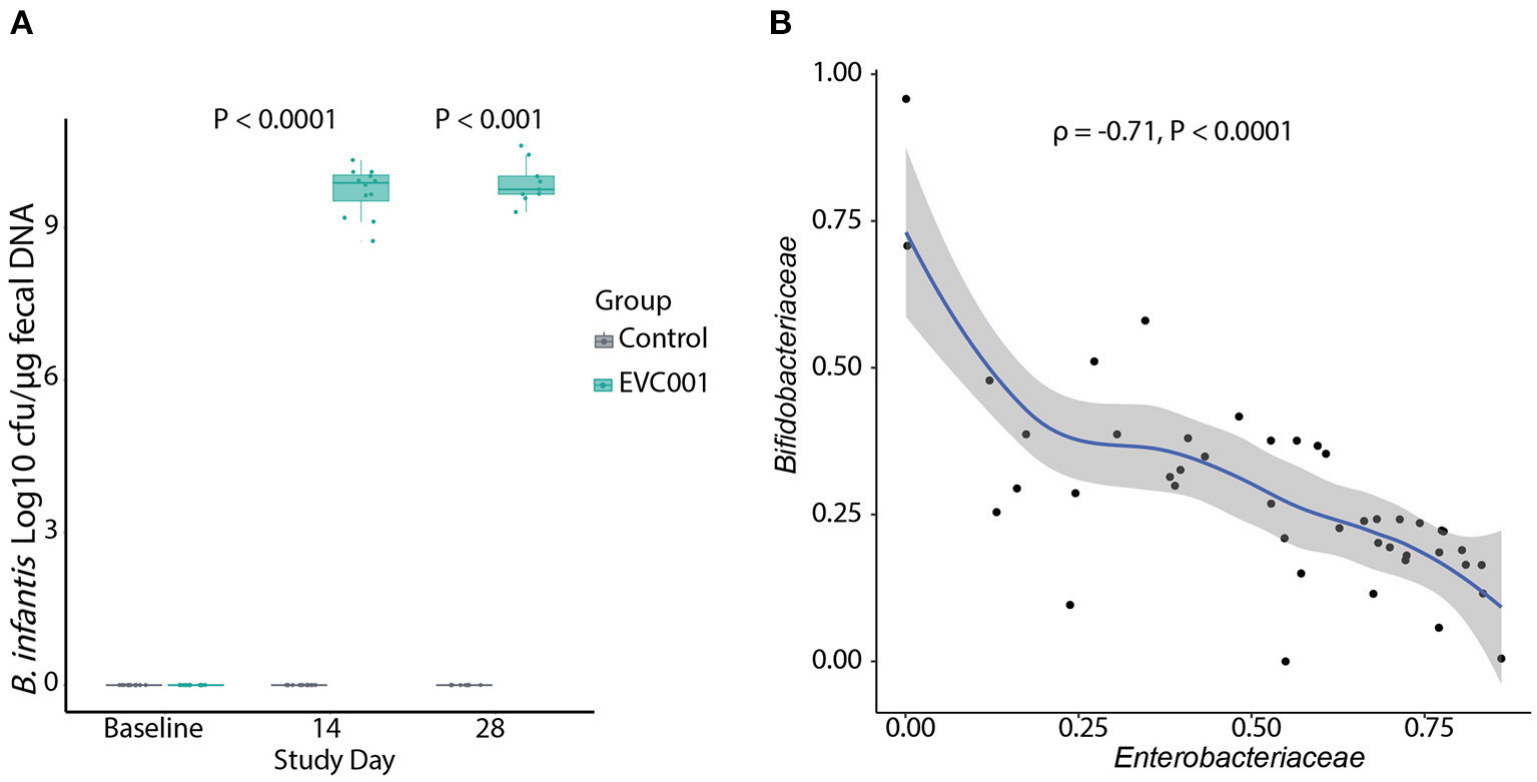
**(A)** Absolute quantification of *B. infantis* by qPCR. The data are represented as Log10 CFU per μg of genomic DNA extracted from swab stool samples. Data in boxplots show the median, first, and third quartiles. Statistical differences determined by Wilcoxon Rank Sum test. **(B)** Negative correlation (Spearman's) between the abundance of *Bifidobacteriaceae* and *Enterobacteriaceae* families in infants in the EVC001 group. Data points for baseline were excluded from the correlation analysis. All *P*-values are FDR-corrected.

*B. infantis* was undetectable in all samples at baseline (Figure 2A). By Study Days 14 and 28, *B. infantis* remained undetectable in infants in the control group while it increased significantly, to an average of 9.7 and 9.9 log10 CFU per μg of fecal DNA at Study Days 14 and 28, respectively, in the fecal samples from infants in the EVC001 group (Figure 2A).

Next, 16S rRNA gene sequencing was used to determine the composition of the fecal microbiota of infants in this study. After quality filtering, an average of 34,314 (SD = 10,312) reads were retained per sample and used for taxonomic comparisons. Samples with fewer than 15,000 reads were removed from analysis, which removed 4 samples and included negative-control samples (PCR and extraction controls). The top ten families comprising the microbiota of infants at baseline were identified as *Bacteroidaceae, Clostridiaceae, Enterobacteriaceae, Enterococcacoae, Peptostreptococcaceae, Staphylococcaceae, Streptococcaceae*, and *Veillonellaceae*. No significant differences in the abundance of the bacterial families were detected between groups at baseline. By Day 14 after the introduction of *B. infantis* EVC001, the *Bifidobacteriaceae* family was significantly increased in the EVC001 group (Wilcoxon, FDR-adjusted *P-*value = 0.01). By Day 28 the *Bifidobacteriaceae* remained higher, compared to control (Wilcoxon, FDR-adjusted *P-*value = 0.06) (Supplemental Table 4). Despite these composition changes, there were no differences in the Shannon index diversity between the EVC001 and control groups at Study Days 14 and 28 (Supplemental Figure 2A). The adonis test indicated a significant difference in overall diversity between the treatment groups (*P* = 0.001, *R*^2^ = 0.308), suggesting supplementation with EVC001 shows a strong effect in driving the beta diversity between groups (Supplemental Figure 2B). Additional testing of weighted unifrac distances stratified by study day showed Group had no significant effect on beta diversity at baseline; compared to the significant effect at Day 14 (*R*^2^ = 0.339, *P* = 0.001) and Day 28 (*R*^2^ = 0.316, *P* = 0.003). Overall, binary Jaccard distances were increased in the subjects of the control group when compared to their baseline sample over time (*P* = 0.038) (Supplemental Figure 2C). We explored correlations between taxa and found the relative abundance of *Bifidobacteriaceae* was strongly and negatively correlated with *Enterobacteriaceae* abundance (Figure 2B), including genera associated with negative outcomes in preterm infants such as *Klebsiella* and *Escherichia*. Together, these findings indicate the strain *B. infantis* EVC001 can colonize the preterm infant gut in high numbers.

### HMO Utilization

**Figure 3:**
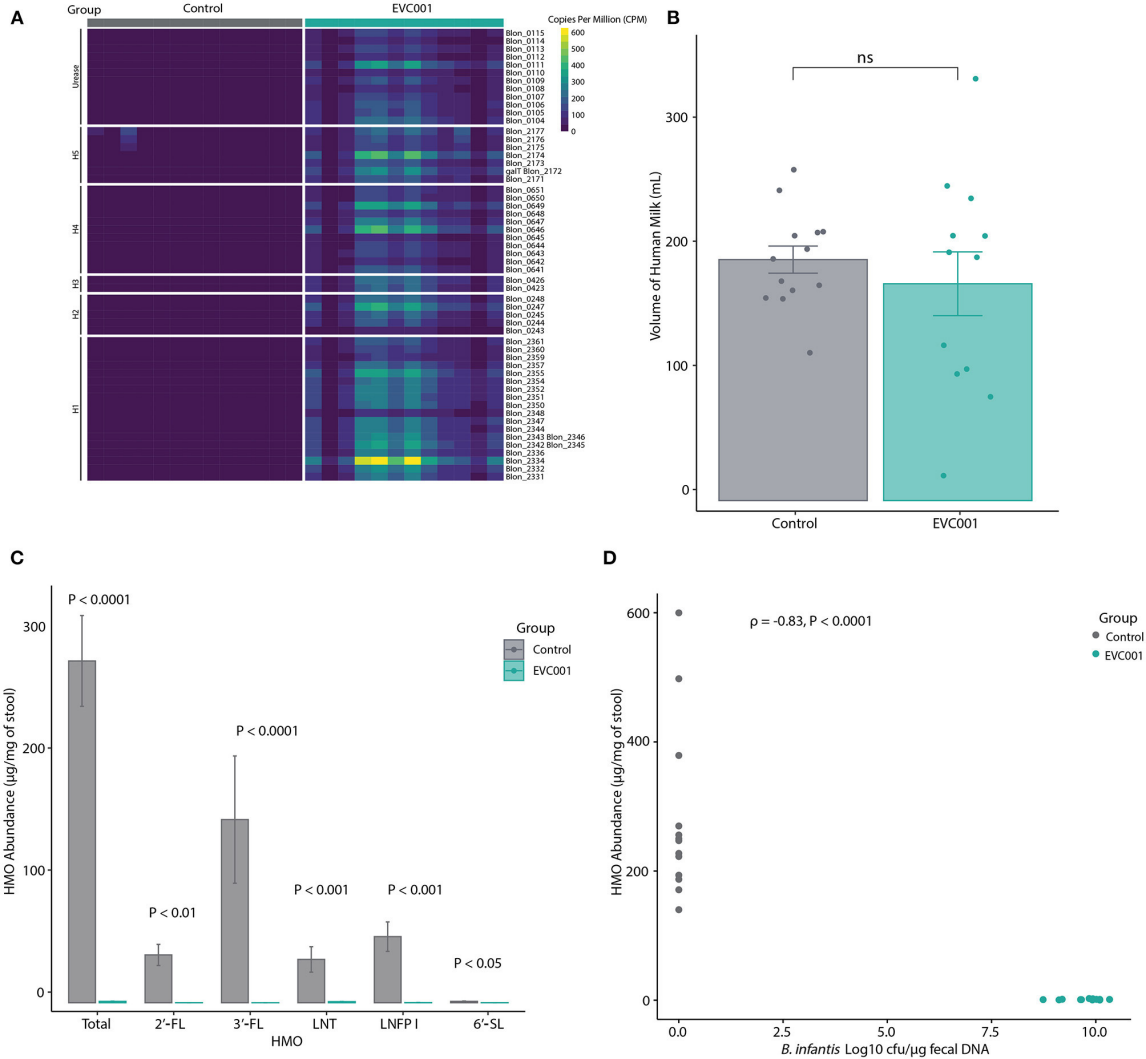
For all panels, all available samples were analyzed from Study Day 14 [*n* = 13 control, *n* = 12 EVC001]. **(A)** Heatmap showing the relative abundance of HMO-utilization genes in the fecal metagenomes of preterm infants in the control (gray boxes) and EVC001 (teal boxes) groups. As denoted, rows represent genes within in the urease operon, a 43-kb cluster associated with utilization of human milk oligosaccharides (H-1), putative fucose utilization regions (H-2 and H-3), putative sialic acid utilization region (H-4) and LNB metabolism gene region (H-5). **(B)** Average and standard error of volume of human milk consumed by infants in the control and EVC001 groups. Individual data points are represented. ns, not significant. **(C)** Absolute abundance and standard error of HMOs in the fecal samples. Statistical differences determined with Wilcoxon Rank Sum test. **(D)** Spearman's correlation between abundance of HMOs and the absolute abundance (Log10 CFU) of *B. infantis* in the fecal samples. Data points represent individual infants and are colored by group.

*B. infantis* EVC001 was chosen for this study as it contains all genes required to metabolize HMOs from human milk. These functions are located in 5 distinct gene clusters (numbered H1 to H5) in addition to the urease metabolism cluster (21, 45). Thus, the presence of these HMO-utilization genes in fecal metagenomes from the infants in this study was assessed. As shown in Figure 3A and Supplemental Table 5, all HMO-utilization genes were generally abundant in the metagenomes of infants in the EVC001 group but absent in the control group. To determine if the presence of HMO-utilization genes in the fecal metagenome was related to the functional ability of the microbiota to metabolize HMOs, the absolute abundance (μg/mg) of HMOs in the fecal samples was measured. Despite having similar intakes of human milk (control = 194 mL SE 39.5 mL, EVC001 = 174 mL SE 88; *P* = 0.61) (Figure 3B), infants in the EVC001 group excreted significantly less HMOs in their stool compared to infants in the control group. The mean of total measured HMOs in fecal samples of the control infants was 28.01 μg/mg (SE = 3.72) and 0.11 μg/mg (SE = 0.02) in the EVC001 control, a nearly 250-fold difference (Figure 3C). Abundant HMOs in human milk such as 2'FL, 3-FL and 6'SL were below detection limits in the samples from infants in the EVC001 group. Importantly, the abundance of HMOs in the fecal samples were negatively correlated to the absolute abundance of *B. infantis* (Figure 3D). Furthermore, the abundance of ortholog genes (KOs) involved in the uptake, degradation, and metabolism of HMO were driven by the abundance of *B. longum* (inclusive of *B. longum* subsp. *infantis*). In the EVC001 group, all functions related to lacto-*N*-biose metabolism, fucosylated and sialylated HMO degradation were driven entirely by *B. longum* (Supplemental Figure 3). Taken together, *B. infantis* in the EVC001 group provided the microbiota the capacity to metabolize HMOs, which resulted in better metabolism of these molecules in the preterm infant gut.

## Discussion

Anatomical and immunological immaturity of the gut, as well as exposure to antibiotics and the NICU environment, leads to altered bacterial colonization of the preterm gut, referred to as dysbiosis. Colonization with bifidobacteria in particular, is significantly delayed in preterm infants (18, 46). In previous studies, supplementation with *B. infantis* EVC001 has been shown to increase the abundance of total bifidobacteria, resulting in positive effects including reduced abundance of enterobacteria and decreased signs of enteric inflammation in both term and preterm infants (17, 18, 47); however, safety and tolerability of EVC001 in a preterm population had yet to be specifically assessed in a controlled study with prospective adverse event data collection. The study product was fed to preterm infants for a wide range of feedings, with the most premature infants receiving up to 100 feeds of the product. This assessment is particularly important as, despite evidence reporting infants supplemented with probiotics have better outcomes (48), safety concerns continue to be one major hurdle for the clinical use of probiotics in the NICU as part of standard feeding protocols (49).

Results of this study indicate that *B. infantis* EVC001 was well-tolerated and safely consumed in this limited sample size of preterm infants. All reported adverse events were typical of infants of premature age and the incidences were not significantly increased in the EVC001 group. Importantly, no cases of sepsis were reported in the EVC001 group and GI-related events were unchanged. There was a significant reduction in the number of infants with a diagnosis of anemia after the baseline period in the EVC001 group compared to the control group (*P* = 0.02). Of the safety and tolerability endpoints, only the average number of stools per day was significantly different between groups at baseline. Upon supplementation, the number of stools per day decreased significantly in the EVC001 group compared to a significant increase in controls. This observation is consistent with previous reports in term infants consuming *B. infantis* EVC001 in which the frequency of stooling was also reduced compared to the controls (50). It is important to note the decrease in stooling frequency did not result in increased use of glycerin suppositories and/or motility medications, suggesting changes in the stooling frequency did not lead to constipation but was rather related to better metabolism of human milk components (discussed below). Another factor related to stooling was the incidence of diaper dermatitis, which was reported in two infants in the control group but for none in the EVC001 group. Although differences in diaper dermatitis were not statistically significant, previous reports in larger numbers of preterm infants support a reduction in this common skin affliction for infants that receive EVC001 as standard of care (18). These changes in stooling and diaper dermatitis could be related to the improved metabolism of HMOs and the resulting biochemical changes in the gut milieu.

Human milk contains an abundant (up to 15% of macronutrients) and diverse array of HMOs indigestible by human enzymes. Of the bifidobacteria, *B. infantis* has been proven repeatedly to be specifically adapted to metabolize HMOs and therefore to be evolutionarily adapted to colonize the intestine of human milk-fed infants (21). Using subspecies-specific qPCR assays, it was confirmed that the strain EVC001 was able to universally colonize all infants in the EVC001 group. As expected, *B. infantis* was not found to be carried by any infants in the control group. Given the proven ability of this strain to metabolize HMOs, it was hypothesized that infants in the EVC001 group would excrete less HMOs in their stool compared to their control counterparts. Results showed that indeed, infants in the EVC001 had almost undetectable levels of HMOs in their stool, suggesting complete utilization in the gut. These results are relevant, as recent data suggest metabolites found in the fecal samples of term infants exclusively breastfed and colonized with EVC001 can directly influence the development of the immune system (3). Furthermore, preterm infants fed EVC001 with a higher representation of genes required to metabolize HMOs in their metagenome (similarly to infants in this study) have significantly reduced signs of chronic enteric inflammation (18), which may explain why infants receiving EVC001 as standard of care have a significantly lower incidence of NEC and a significantly reduced mortality from NEC (51). Metabolism of HMOs by *B. infantis* results in the formation of organic acids, mainly lactic and acetic acids, which lowers the fecal pH. The acidified gut milieu is both deterrent to the proliferation of detrimental bacteria including known pathogens (4, 5) as well as a deactivator for fecal enzymes implicated in the breakdown of skin, leading to diaper dermatitis (52). Therefore, the results generated from this study and from previous studies utilizing EVC001 support that this strain provides infants with the capacity to metabolize HMOs with positive results in immune development, as well as decreasing the risk of developing co-morbidities common to prematurity and related to enteric inflammatory conditions, such as NEC (51). While it is widely accepted that preterm infants benefit from human milk, here we demonstrate the value of matching the right bacteria with diet. This symbiotic approach to care in the NICU has important implications for stabilizing the gut by fostering the establishment of an infant-appropriate gut microbiota to maximize nutrient acquisition from human milk.

Finally, results from this study are consistent with previous reports of dysbiosis in preterm infants, including high relative abundance of *Enterobacteriaceae* and absent or low abundance of *Bifidobacteriaceae*, despite all infants being fed human milk. In fact, the lower abundance of bifidobacteria found in control infants indicates human milk alone is not sufficient to encourage higher levels of bifidobacteria in the preterm gut. EVC001 resulted in significantly increased abundance of *Bifidobacteriaceae*. Other studies supplementing with *Bifidobacterium* species and strains have shown increases in the relative abundance of this family (53), but the effects on HMO utilization have not been tested. Given infants in the control group had detectable levels of *Bifidobacteriaceae* (identified as *B. breve*), but not *B. infantis*, these results suggest that the presence of *B. infantis* specifically is required for the complete metabolism of HMOs. Additionally, larger Jaccard distances in the control group over time may suggest there is higher species turnover in the control group. This indicates EVC001 supplementation provides a more stable gut community, which is consistent with previous studies (15).

One strength of this study is that potential confounding variables including sex, birth weight, gestational age at birth and mode of delivery were not statistically different between groups. Furthermore, apart from the study intervention, all standard of care practices were equivalent between groups. Thus, the results observed can be reliably attributed to the effects of EVC001. However, this study is not without limitations. First, the control arm did not receive a placebo and treatment arms were not blinded. The decision to conduct the study using a prospective, open-label, sequenced group design was intentional in order to prevent potential cross-colonization of the control infants, since cross-colonization into the placebo arm has been reported in previous trials using powdered preparations of *Bifidobacterium* (20). *B. infantis* EVC001 has been supplemented to hundreds of human milk-fed infants in human clinical trials and consistently colonizes to high levels without adverse events (17, 18). This, along with research regarding transfer of microbes within the hospital environment (10, 46), specifically in the NICU, supports the likely outcome of cross-colonization in an RCT. To minimize the influence of investigator bias in light of the selected study design, on-site investigators were independent of the study sponsor; furthermore, financial compensation to study investigators was agnostic of study outcomes. Finally, all biological samples were de-identified prior to analysis, thus further reducing the potential for investigator bias. The relatively small sample size limits the statistical power of the analysis, particularly with respect to clinical outcomes. While the results are supportive, continued evaluation of adverse events related to the product in larger numbers of infants (specifically for extremely rare events such as probiotic sepsis) will further contribute to the evidence base for overall safety and tolerability of *B. infantis* EVC001. Nonetheless, most, if not all microbiota and HMO-utilization results generated here are supported by previous observational trials in similar populations as well as *in vitro* data to support mechanisms of action (3, 18, 21).

In this study, we did not observe any safety concerns with the use of *B. infantis* EVC001 in MCT oil in preterm infants over a wide range of prematurity states receiving up to 100 daily feeds of the product. Importantly, gut colonization with activated *B. infantis* EVC001 resulted in a significantly increased bifidobacteria population with a concomitant increased capacity to metabolize key nutrients (i.e., HMOs) from human milk. Given provision of human milk is almost universally standard of care across NICUs in the US, providing *B. infantis* EVC001 may provide several benefits through unlocking the full potential of human milk's nutritional value as demonstrated in previous studies using this strain.

## Data Availability Statement

The datasets presented in this study can be found in online repositories. The names of the repository/repositories and accession number(s) can be found below: https://www.ncbi.nlm.nih.gov/, PRJNA772730.

## Ethics Statement

The studies involving human participants were reviewed and approved by Orlando Health Institutional Review Board. Written informed consent to participate in this study was provided by the participant's legal guardian/next of kin.

## Author Contributions

OC was involved in the design and implementation of research. SB, MC, SN, CM, KW, and JP conducted research. AA, AM, EK, and RM analyzed data. SB and RD contributed equally to writing the paper. All authors read and approved the final manuscript.

## Conflict of Interest

RM, OC, and RD are employed by Evolve BioSystems, Inc. AA, AM, and EK are employees of Clarity Genomics, Inc., and Arome Science Inc., and were compensated by Evolve BioSystems, Inc. for their contributions to the statistical analysis. SB, MC, CM, KW, SN, and JP were study personnel at Orlando Health Winnie Palmer Hospital, which was funded by Evolve BioSystems, Inc.

## Publisher's Note

All claims expressed in this article are solely those of the authors and do not necessarily represent those of their affiliated organizations, or those of the publisher, the editors and the reviewers. Any product that may be evaluated in this article, or claim that may be made by its manufacturer, is not guaranteed or endorsed by the publisher.

## Supplementary Material

Supplemental Figure 1 Participant flow chart indicating infants selected for inclusion in analyses.

Supplemental Figure 2 **(A)** Shannon index diversity and **(B)** beta diversity (Weighted Unifrac) between the EVC001 and control groups at Study Days 14 [*n* = 13 control, *n* = 12 EVC001] and 28 [*n* = 9 control, *n* = 10 EVC001]. Treatment group effect size and significance shown. **(C)** Binary Jaccard distances from baseline sample to Study Days 14 and 28 between EVC001 and control groups.

Supplemental Figure 3 Relative abundance (CPM) and functional annotation of KEGG orthologous genes related to HMO-utilization mapped to HMO utilization taxa *Bifidobacterium breve*, or *B. longum* (inclusive of *B. infantis*) at Study Day 14 [*n* = 13 control, *n* = 12 EVC001].
