# Supplementary figures and images for: B. infantis EVC001 Is Well-Tolerated and Improves Human Milk Oligosaccharide Utilization in Preterm Infants in the Neonatal Intensive Care Unit
Source: Front Pediatr. 2022 Jan 5;9:795970. doi: 10.3389/fped.2021.795970 (PMC8767116; doi:10.3389/fped.2021.795970)

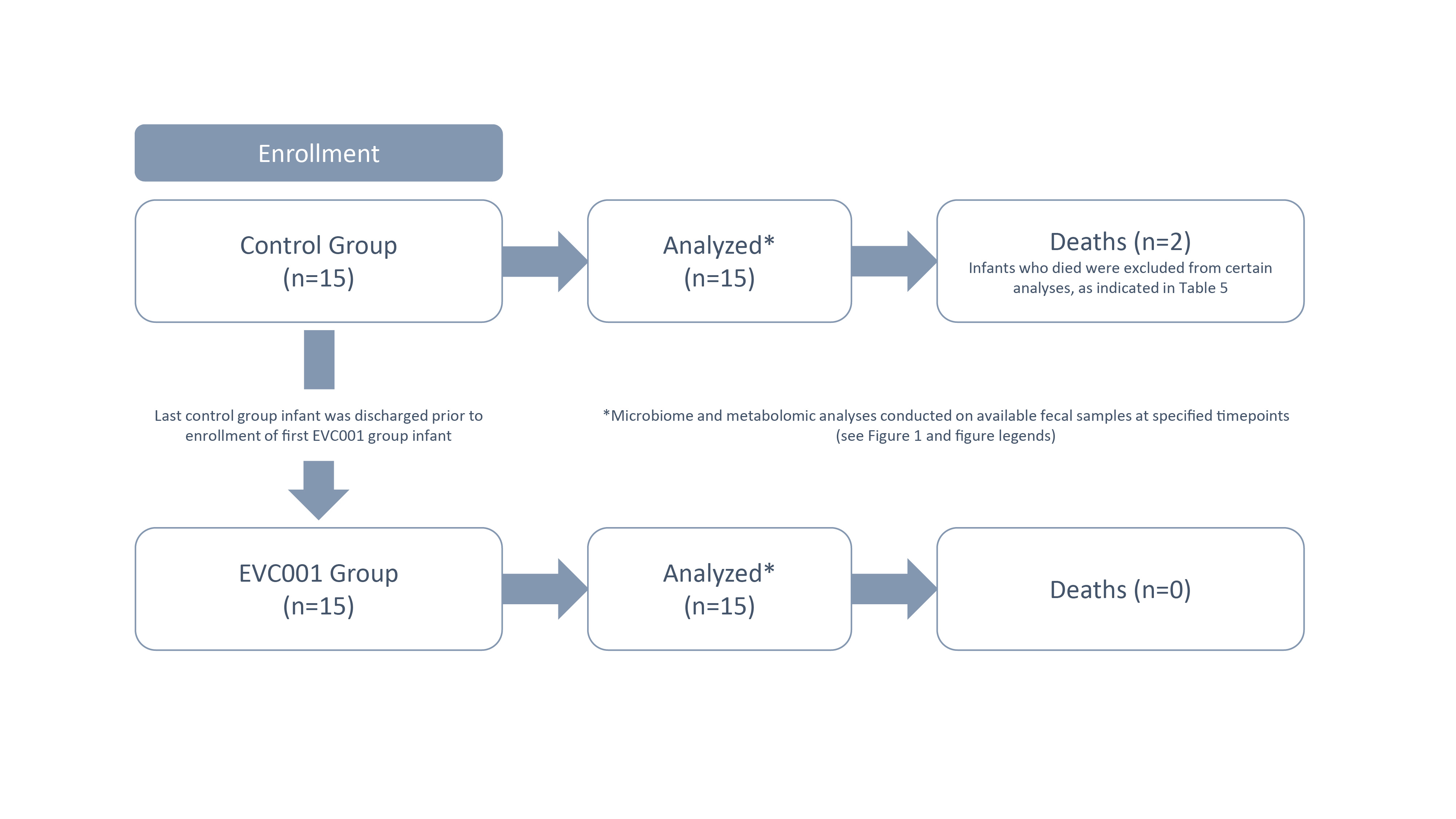

Supplement: Supplemental Figure 1 — Participant flow chart indicating infants selected for inclusion in analyses. [file Image_1.jpg]

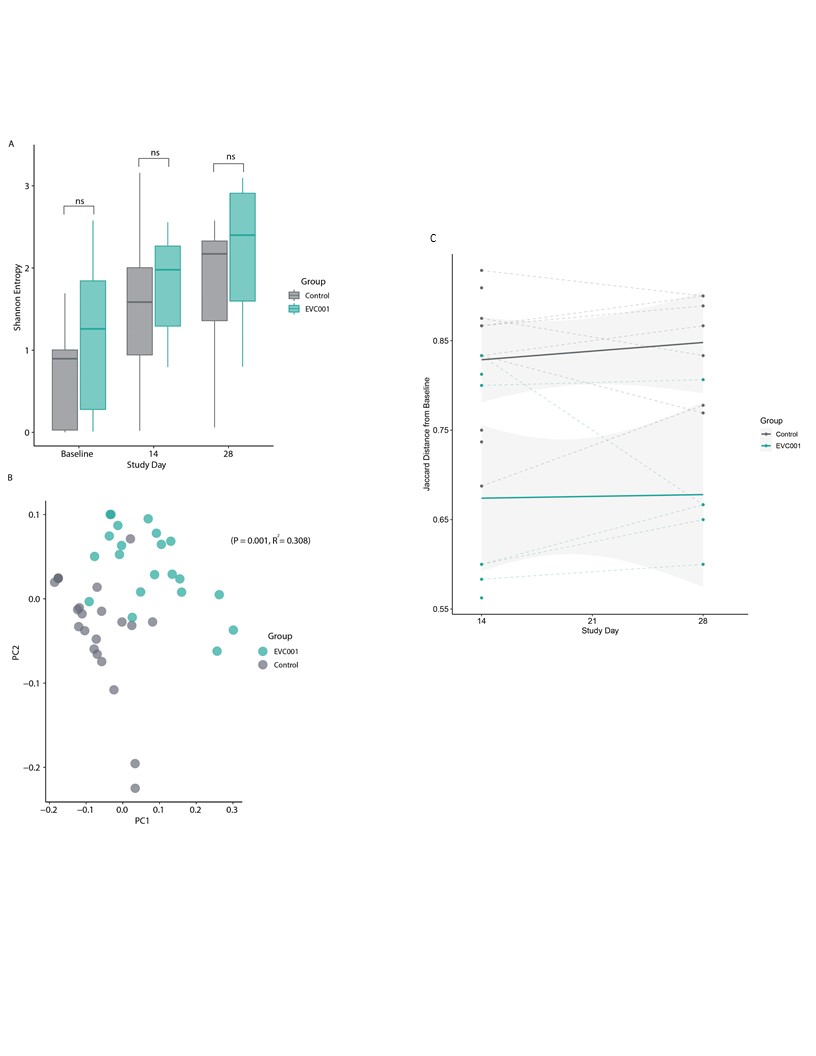

Supplement: Supplemental Figure 2 — (A) Shannon index diversity and (B) beta diversity (Weighted Unifrac) between the EVC001 and control groups at Study Days 14 [n = 13 control, n = 12 EVC001] and 28 [n = 9 control, n = 10 EVC001]. Treatment group effect size and significance shown. (C) Binary Jaccard distances from baseline sample to Study Days 14 and 28 between EVC001 and control groups. [file Image_2.jpeg]

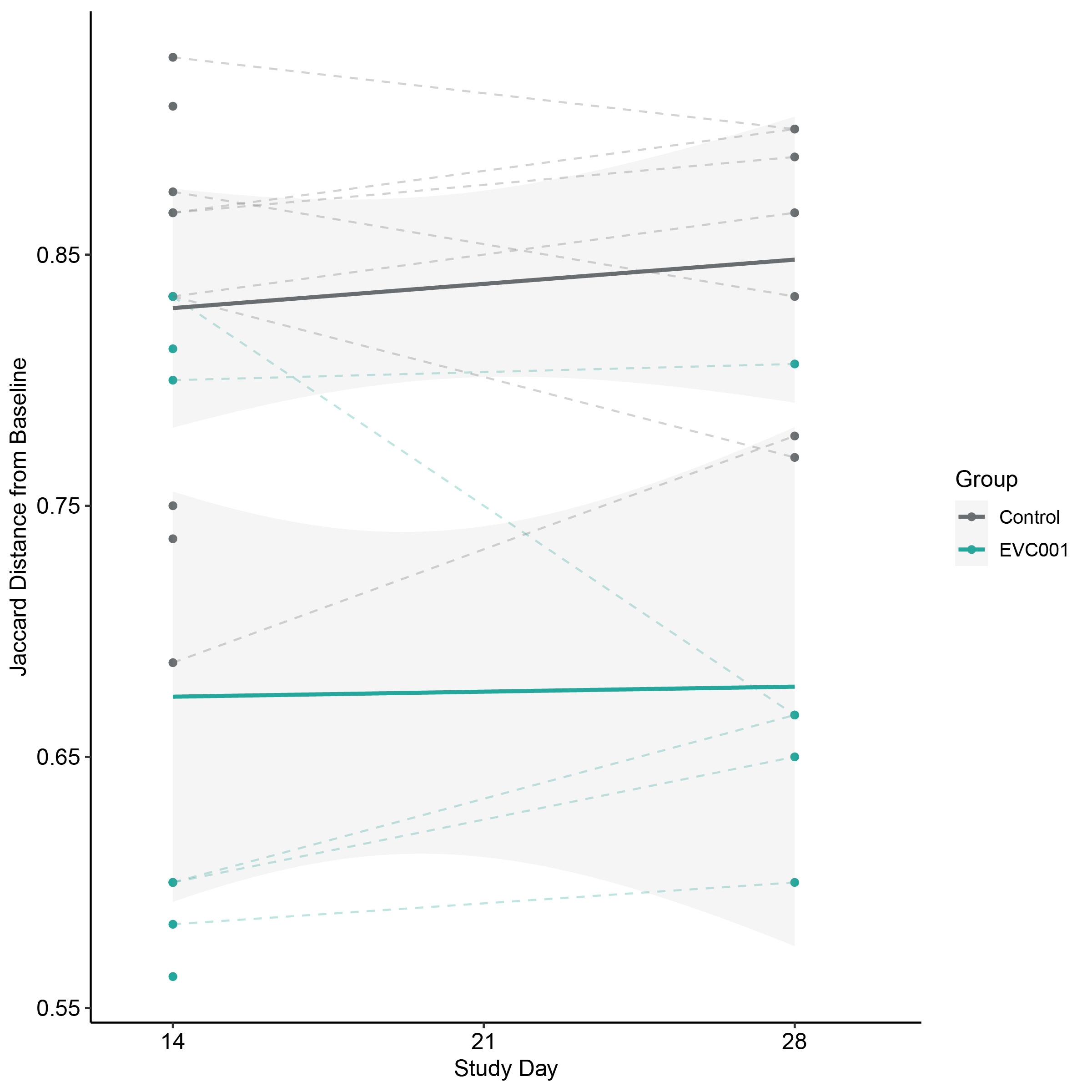

Supplement: Supplemental Figure 3 — Relative abundance (CPM) and functional annotation of KEGG orthologous genes related to HMO-utilization mapped to HMO utilization taxa Bifidobacterium breve, or B. longum (inclusive of B. infantis) at Study Day 14 [n = 13 control, n = 12 EVC001]. [file Image_3.jpg]

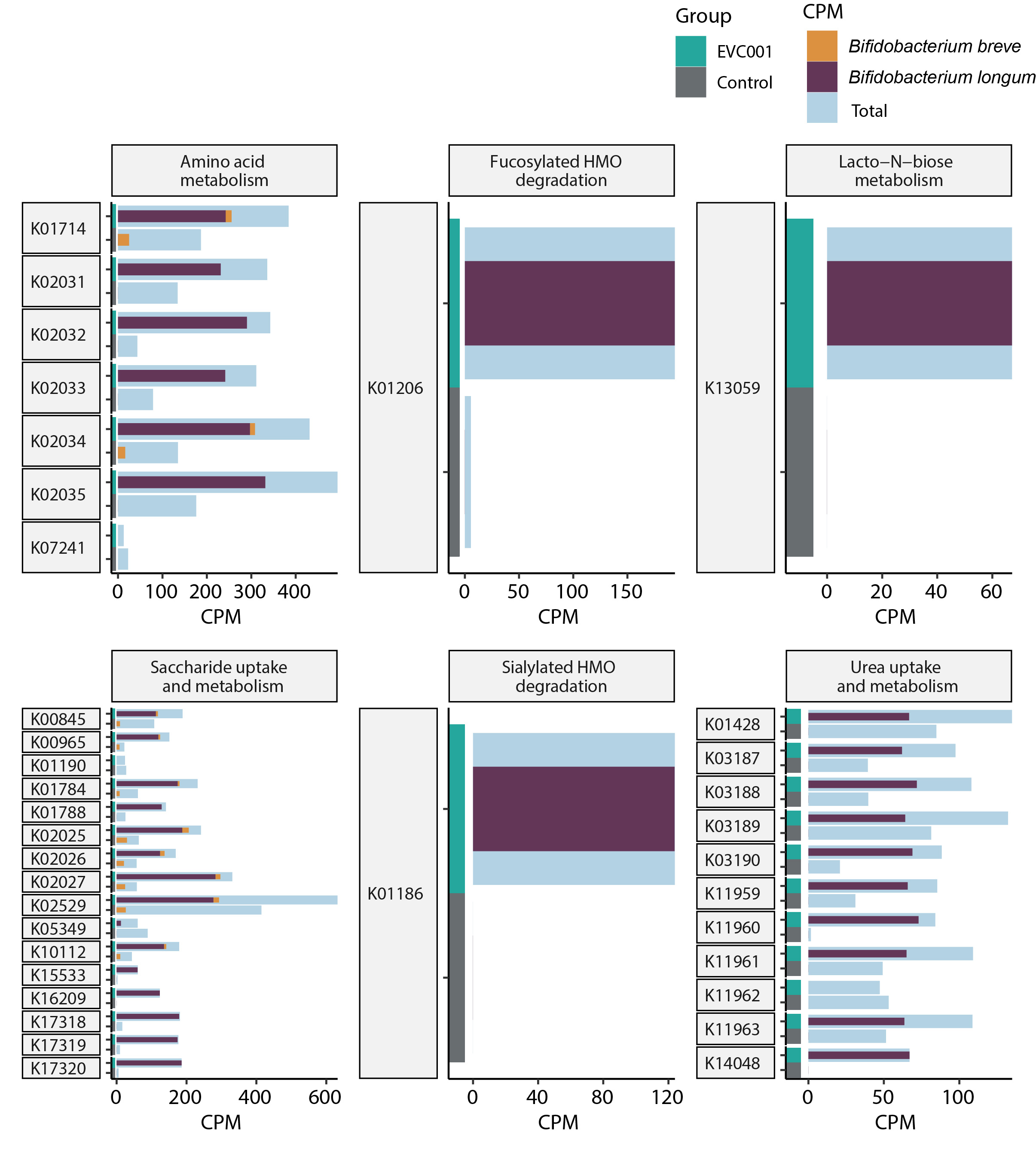

Supplement: Supplementary file 4 [file Image_4.jpg]

Supplemental Table 4: 16S rRNA Gene Sequencing Differential Abundance Analysis


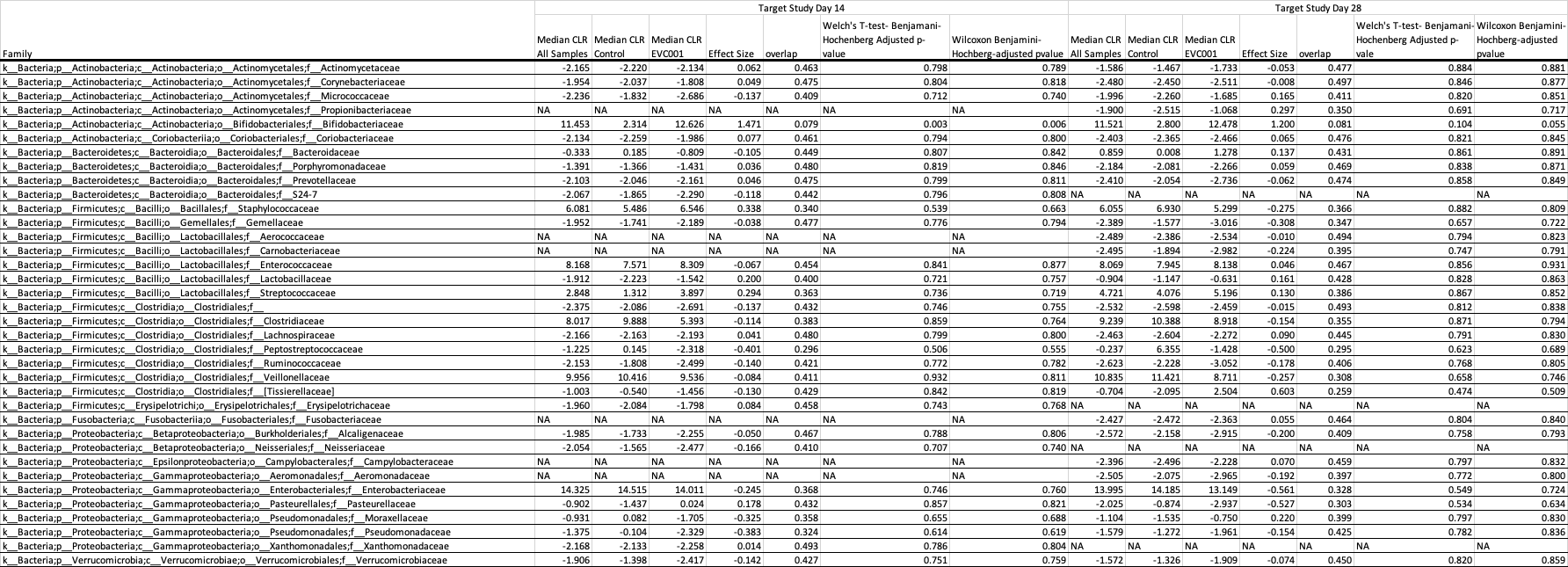

Supplement: Supplementary file 8 [file Table_4.docx]
