# Supplementary material for: B. infantis EVC001 Is Well-Tolerated and Improves Human Milk Oligosaccharide Utilization in Preterm Infants in the Neonatal Intensive Care Unit
Source: Front Pediatr. 2022 Jan 5;9:795970. doi: 10.3389/fped.2021.795970 (PMC8767116; doi:10.3389/fped.2021.795970)
Supplement: Supplementary file 5 [file Table_1.docx]

Supplemental Table 1: Baseline Clinical Data

|  |  | Control Group (n=15) | | EVC001 Group (n=15) | | |  |
| --- | --- | --- | --- | --- | --- | --- | --- |
| **Diagnosis** |  | **Count** | **Percent** | | **Count** | **Percent** | ***P*-value**^†^ |
|  | Anemia | 0 | 0% | | 1 | 7% | 1.00 |
|  | Feeding intolerance | 0 | 0% | | 1 | 7% | 1.00 |
|  | Hyperbilirubinemia prematurity | 7 | 47% | | 11 | 73% | 0.26 |
|  | Hypocalcemia | 0 | 0% | | 1 | 7% | 1.00 |
|  | Hypoglycemia | 1 | 7% | | 0 | 0% | 1.00 |
|  | Hyponatremia | 1 | 7% | | 0 | 0% | 1.00 |
|  | Intrauterine Growth Restriction | 1 | 7% | | 0 | 0% | 1.00 |
|  | Pulmonary Hypertension | 1 | 7% | | 0 | 0% | 1.00 |
|  | Respiratory Distress Syndrome | 1 | 7% | | 0 | 0% | 1.00 |
|  | Suspected sepsis / Rule out sepsis | 8 | 53% | | 5 | 33% | 0.46 |
|  | Thrombocytopenia | 1 | 7% | | 0 | 0% | 1.00 |
|  | Twin to twin transfusion | 1 | 7% | | 0 | 0% | 1.00 |
| ^†^ Fisher’s exact test | | | | | | | |
