# Supplementary material for: B. infantis EVC001 Is Well-Tolerated and Improves Human Milk Oligosaccharide Utilization in Preterm Infants in the Neonatal Intensive Care Unit
Source: Front Pediatr. 2022 Jan 5;9:795970. doi: 10.3389/fped.2021.795970 (PMC8767116; doi:10.3389/fped.2021.795970)
Supplement: Supplementary file 6 [file Table_2.docx]

Supplemental Table 2: Other Outcomes of Interest

|  |  | | Control Group (n=15) | EVC001 Group (n=15) |  | |
| --- | --- | --- | --- | --- | --- | --- |
| **Outcome** | |  | **Mean** | **Mean** | ***P*-value**^‡^ | |
|  | Study Day 0 (day of life) | | 5.7 | 9 | 0.0003 | |
|  | Rate of weight gain (g/kg/day) | | 16^Ϯ^ | 15 | 0.59 | |
|  | Achieved weight (g) | | 2458^Ϯ^ | 2599 | 0.93 | |
|  | Achieved length (cm) | | 45.15^Ϯ^ | 45.12 | 0.61 | |
|  | Achieved head circumference (cm) | | 32.36^Ϯ^ | 32.87 | 0.63 | |
|  | Time to full feeds at 150 mLs/kg/day (days) | | 7.6^Ϯ^ | 6.9 | 0.93 | |
|  | Stools/day at baseline (#) | | 2.46 | 3.64 | 0.0003 | |
|  | Stools/day post-baseline (#) | | 4.48 | 2.68 | 0.002 | |
| ^‡^ Wilcoxon rank sum test  ^Ϯ^ infants who died (n=2) not included | | | | | |  |
