# Supplementary material for: B. infantis EVC001 Is Well-Tolerated and Improves Human Milk Oligosaccharide Utilization in Preterm Infants in the Neonatal Intensive Care Unit
Source: Front Pediatr. 2022 Jan 5;9:795970. doi: 10.3389/fped.2021.795970 (PMC8767116; doi:10.3389/fped.2021.795970)
Supplement: Supplementary file 7 [file Table_3.docx]

|  | Control Group (n=15) | | EVC001 Group (n=15) | |  | |
| --- | --- | --- | --- | --- | --- | --- |
|  | **Count** | **Percentage** | **Count** | **Percentage** | | ***P-*value**^†^ |
| Caffeine | 12 | 80% | 8 | 53% | | 0.25 |
| Systemic Antibiotics | 8 | 53% | 5 | 33% | | 0.46 |
| Antifungals | 1 | 7% | 0 | 0% | | 1 |
| H2 Blockers / PPIs | 0 | 0% | 0 | 0% | | 1 |
| Enemas / Suppositories / Motility Medications | 2 | 13% | 2 | 13% | | 1 |
| ^†^ Fisher’s exact test | | | | | | |

Supplemental Table 3: Concomitant Medications
