# Supplementary material for: B. infantis EVC001 Is Well-Tolerated and Improves Human Milk Oligosaccharide Utilization in Preterm Infants in the Neonatal Intensive Care Unit
Source: Front Pediatr. 2022 Jan 5;9:795970. doi: 10.3389/fped.2021.795970 (PMC8767116; doi:10.3389/fped.2021.795970)
Supplement: Supplementary file 9 [file Table_5.docx]

Supplemental Table 5: HMO Genes at Day 14

| HMO Gene | Control Group n | Control Mean CPM | Control SD CPM | EVC001 Group n | EVC001 Mean CPM | EVC001 SD CPM | *P*-value |
| --- | --- | --- | --- | --- | --- | --- | --- |
| Blon_0104 | 13 | 0 | 0 | 12 | 130.531 | 78.859 | <0.0001 |
| Blon_0105 | 13 | 0 | 0 | 12 | 92.502 | 56.199 | <0.0001 |
| Blon_0106 | 13 | 0 | 0 | 12 | 108.318 | 64.979 | <0.0001 |
| Blon_0107 | 13 | 0 | 0 | 12 | 77.397 | 47.449 | <0.0001 |
| Blon_0108 | 13 | 0 | 0 | 12 | 61.564 | 36.717 | <0.0001 |
| Blon_0109 | 13 | 0 | 0 | 12 | 94.112 | 54.86 | <0.0001 |
| Blon_0110 | 13 | 0 | 0 | 12 | 63.449 | 37.612 | <0.0001 |
| Blon_0111 | 13 | 0 | 0 | 12 | 180.535 | 108.158 | <0.0001 |
| Blon_0112 | 13 | 0 | 0 | 12 | 55.282 | 32.381 | <0.0001 |
| Blon_0113 | 13 | 0 | 0 | 12 | 83.293 | 50.245 | <0.0001 |
| Blon_0114 | 13 | 0 | 0 | 12 | 51.12 | 30.517 | <0.0001 |
| Blon_0115 | 13 | 0 | 0 | 12 | 82.326 | 49.559 | <0.0001 |
| Blon_0243 | 13 | 0.634 | 2.26 | 12 | 42.402 | 25.185 | <0.0001 |
| Blon_0244 | 13 | 1.641 | 5.562 | 12 | 94.751 | 56.553 | <0.0001 |
| Blon_0245 | 13 | 2.128 | 7.465 | 12 | 113.343 | 67.723 | <0.0001 |
| Blon_0247 | 13 | 2.238 | 7.863 | 12 | 196.957 | 119.132 | <0.0001 |
| Blon_0248 | 13 | 1.526 | 5.297 | 12 | 130.32 | 78.275 | <0.0001 |
| Blon_0423 | 13 | 0 | 0 | 12 | 113.316 | 68.427 | <0.0001 |
| Blon_0426 | 13 | 1.075 | 3.729 | 12 | 130.536 | 78.244 | <0.0001 |
| Blon_0641 | 13 | 0 | 0 | 12 | 93.534 | 58.296 | <0.0001 |
| Blon_0642 | 13 | 0 | 0 | 12 | 54.815 | 34.233 | <0.0001 |
| Blon_0643 | 13 | 0 | 0 | 12 | 81.581 | 50.121 | <0.0001 |
| Blon_0644 | 13 | 0 | 0 | 12 | 72.536 | 45.627 | <0.0001 |
| Blon_0645 | 13 | 0 | 0 | 12 | 55.689 | 35.158 | <0.0001 |
| Blon_0646 | 13 | 0 | 0 | 12 | 218.174 | 135.357 | <0.0001 |
| Blon_0647 | 13 | 0 | 0 | 12 | 141.405 | 88.071 | <0.0001 |
| Blon_0648 | 13 | 0 | 0 | 12 | 84.142 | 52.215 | <0.0001 |
| Blon_0649 | 13 | 0.006 | 0.021 | 12 | 186.982 | 115.331 | <0.0001 |
| Blon_0650 | 13 | 0 | 0 | 12 | 69.992 | 44.667 | <0.0001 |
| Blon_0651 | 13 | 0 | 0 | 12 | 77.102 | 48.606 | <0.0001 |
| Blon_2171 | 13 | 4.532 | 10.13 | 12 | 101.439 | 58.813 | <0.0001 |
| Blon_2173 | 13 | 0 | 0 | 12 | 107.787 | 64.759 | <0.0001 |
| Blon_2174 | 13 | 3.843 | 8.727 | 12 | 224.13 | 133.849 | <0.0001 |
| Blon_2175 | 13 | 5.142 | 12.664 | 12 | 81.949 | 46.624 | <0.0001 |
| Blon_2176 | 13 | 10.329 | 25.136 | 12 | 94.652 | 54.346 | <0.0001 |
| Blon_2177 | 13 | 16.158 | 37.353 | 12 | 129.431 | 72.883 | <0.0001 |
| Blon_2331 | 13 | 0 | 0 | 12 | 135.71 | 80.925 | <0.0001 |
| Blon_2332 | 13 | 0 | 0 | 12 | 157.993 | 96.496 | <0.0001 |
| Blon_2334 | 13 | 0.004 | 0.012 | 12 | 319.547 | 193.672 | <0.0001 |
| Blon_2336 | 13 | 0 | 0 | 12 | 149.847 | 91.43 | <0.0001 |
| Blon_2342 Blon_2345 | 13 | 0 | 0 | 12 | 184.315 | 111.098 | <0.0001 |
| Blon_2343 Blon_2346 | 13 | 0.002 | 0.008 | 12 | 165.711 | 99.833 | <0.0001 |
| Blon_2344 | 13 | 0 | 0 | 12 | 151.479 | 90.395 | <0.0001 |
| Blon_2347 | 13 | 0.001 | 0.004 | 12 | 151.339 | 90.563 | <0.0001 |
| Blon_2348 | 13 | 0 | 0 | 12 | 57.37 | 34.212 | <0.0001 |
| Blon_2350 | 13 | 0 | 0 | 12 | 141.184 | 83.044 | <0.0001 |
| Blon_2351 | 13 | 0 | 0 | 12 | 145.444 | 85.174 | <0.0001 |
| Blon_2352 | 13 | 0 | 0 | 12 | 150.561 | 89.082 | <0.0001 |
| Blon_2354 | 13 | 0 | 0 | 12 | 142.674 | 84.001 | <0.0001 |
| Blon_2355 | 13 | 0 | 0 | 12 | 196.866 | 115.326 | <0.0001 |
| Blon_2357 | 13 | 0 | 0 | 12 | 123.48 | 73.431 | <0.0001 |
| Blon_2359 | 13 | 0 | 0 | 12 | 67.931 | 39.979 | <0.0001 |
| Blon_2360 | 13 | 0 | 0 | 12 | 86.403 | 51.819 | <0.0001 |
| Blon_2361 | 13 | 0 | 0 | 12 | 108.303 | 64.034 | <0.0001 |
| galT Blon_2172 | 13 | 0 | 0 | 12 | 156.299 | 95.596 | <0.0001 |
